# Incidence of Influenza-related Medical Encounters and the Associated Healthcare Resource Use and Complications Across Adult Age Groups in the United States During the 2015–2020 Influenza Seasons

**DOI:** 10.1093/cid/ciae180

**Published:** 2024-04-03

**Authors:** Ian McGovern, Katherine Cappell, Alina N Bogdanov, Mendel D M Haag

**Affiliations:** CSL Seqirus, Center for Outcomes Research and Epidemiology, Waltham, Massachusetts, USA; Veradigm, Real World Evidence, Chicago, Illinois, USA; Veradigm, Real World Evidence, Chicago, Illinois, USA; CSL Seqirus, Center for Outcomes Research and Epidemiology, Amsterdam, The Netherlands

**Keywords:** Influenza, Incidence, Disease Burden, Antivirals, Antibiotics

## Abstract

**Background:**

Research on influenza burden in adults has focused on crude subgroups with cut-points at 65 years, limiting insight into how burden varies with increasing age. This study describes the incidence of influenza-related outpatient visits, emergency room visits, and hospitalizations, along with healthcare resource use and complications in the aging adult population.

**Methods:**

Individuals aged ≥18 years in the United States were evaluated retrospectively in 5 seasonal cohorts (2015–2020 seasons) in strata of age with 5-year increments. Person-level electronic medical records linked to pharmacy and medical claims were used to ascertain patient characteristics and outcomes. Influenza-related medical encounters were identified based on diagnostic codes (International Classification of Diseases, 10th Edition, codes J09*–J11*).

**Results:**

Incidence of influenza-related outpatient visits was highest among people aged 18–34 years and declined with increasing age. For emergency room visits, incidence tended to be elevated for people aged 18–34 years, relatively stable from 35 through 60, and increased rapidly after age 60 years. Hospitalization incidence remained relatively stable until about 50 years of age and then increased with age. One in 3 patients was diagnosed with pneumonia after hospitalization, regardless of age. Across seasons, age groups, and clinical settings, on average, 40.8% of individuals were prescribed antivirals and 17.2% antibiotics.

**Conclusions:**

Incidence of influenza-related hospitalizations begins to increase around age 50 years rather than the more common cut-point of 65, whereas incidence of outpatient visits was highest among younger adults. Influenza infections frequently led to antiviral and antibiotic prescriptions, underscoring the role influenza vaccination can play in combating antimicrobial resistance.

## Teaser

Changes in influenza incidence generally occur gradually as individuals age, with increases in hospitalization incidence beginning around 50 years of age. Influenza infections frequently result in serious secondary complications such as pneumonia and are associated with frequent antibiotic and antiviral prescriptions.

## Graphical Abstract

**Graphical Abstract:**
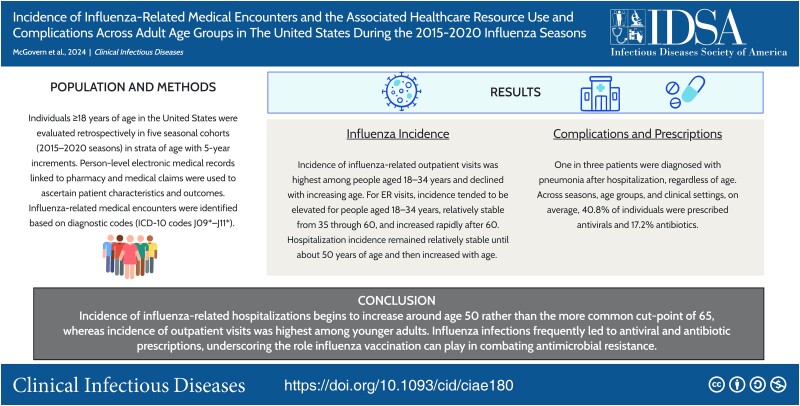
This graphical abstract is also available at Tidbit: https://tidbitapp.io/tidbits/incidence-of-influenza-related-medical-encounters-and-the-associated-healthcare-resource-use-and-complications-across-adult-age-groups-in-the-united-states-during-the-2015-2020-influenza-seasons?utm_campaign=tidbitlinkshare&utm_source=IO

The Centers for Disease Control and Prevention (CDC) estimates that during the 2015–2016 through the 2019–2020 influenza seasons, there were 24–41 million infections, 280 000–710 000 hospitalizations, and 23 000–52 000 deaths annually [1]. The majority of influenza hospitalizations and deaths occur among adults aged ≥65 years [1, 2], and the presence of certain medical conditions can increase the risk of severe outcomes following infection [2–4]. In addition to an increased prevalence of high-risk medical conditions [5], aging is associated with the decline and dysregulation of the immune system, termed immunosenescence [6]. This progressive decline in immune response leads to a decreased ability to respond to infections and develop immunity after vaccination [7, 8]. Although these changes are most noticeable among adults aged ≥65 years, there is no specific age of onset, and it can be exacerbated by genetics, environmental factors, and comorbidities [9].

The focus of influenza interventions and public health initiatives in adults is often among those older than age 65 years or with specific risk factors for influenza; however, there is evidence that individuals between the ages of 50–64 years are also at an elevated risk from influenza infection and severe outcomes following infection [10–12]. This descriptive study aimed to evaluate the incidence of influenza-related outpatient visits, emergency room (ER) visits, and hospitalizations among adult age groups and to evaluate healthcare resource use and complications following influenza-related medical encounters across adult age groups.

## METHODS

### Study Design

We conducted a retrospective, observational cohort study of influenza-related medical encounters and associated healthcare resource use and complications among adults during the 2015–2016, 2016–2017, 2017–2018, 2018–2019, and 2019–2020 influenza seasons. The study was designed, implemented, and reported in accordance with Good Pharmacoepidemiological Practice, applicable local regulations, and the ethical principles in the Declaration of Helsinki [13, 14]. Study findings have been reported according to the Reporting of Studies Conducted using Observational Routinely Collected Health Data recommendations [15].

### Data Sources

We leveraged an integrated dataset of electronic health records (EHRs) sourced from the Veradigm Network EHR linked to closed medical and pharmacy claims data sourced from Komodo Health. The EHR data are sourced from outpatient care facilities, whereas the claims data include outpatient, inpatient, and pharmacy claims. Claims data before 1 January 2015 was not available. For this reason, individuals in the 2015–2016 season may have incomplete baseline data.

This dataset and its application to influenza research have been previously described by Boikos et al [16]. This dataset has been certified as statistically deidentified through a formal determination by a qualified expert as defined in Section §164.514(b)(1) of the Health Insurance Portability and Accountability Act of 1996 Privacy Rule.

Coding systems used in the identification of data variables included International Classification of Diseases, 9th and 10th Edition, Clinical Modification (ICD-9-CM and ICD-10-CM) diagnosis codes, vaccine administration codes, Systematized Nomenclature of Medicine Clinical Terms codes, Current Procedural Terminology codes, and National Drug Codes.

### Study Population

For each influenza season, we identified all individuals who were at least aged 18 years at the start of the season with nonmissing gender and geographic region data. We required that individuals have a record in the EHR during the influenza season and at least 1 year before the start of the season. An influenza season was defined as week 40 of 1 calendar year through week 20 in the subsequent calendar year. In the 2019–2020 season, we truncated the influenza season at the end of week 10 (7 March 2020) to avoid complications in the analysis resulting from the potential widespread circulation of the severe acute respiratory syndrome coronavirus 2 virus. Additionally, we required that individuals be continuously enrolled in claims data from at least 1 year before the start of the season to at least 120 days after the end of the season. Individuals who had an influenza-related medical encounter during the off-season period for that season were excluded (ie, during the 19-week period between the end of the previous influenza season and the start of the subsequent influenza season). Each influenza season was treated as a separate retrospective cohort, with individual patient selection, baseline characteristic assessment, and outcome assessment happening independently for each season, with individuals potentially contributing to 1 or more of the seasonal assessments.

### Cohort Stratifications

This study employed 2 age stratifications. For the main analysis, a refined stratification of 5-year increments of age was used. In addition, the main outcome and all other analyses were assessed in groups stratified by age using broad age ranges (aged 18–49, 50–64, and ≥65 years).

Furthermore, we stratified age groups into risk subgroups based on CDC-identified health conditions that are known to increase a person's risk of experiencing severe influenza (Supplementary Table 1) [4]. Individuals’ medical history was evaluated during a 1-year baseline period that spanned epidemiology week 40 in the calendar year preceding the start of the influenza season through week 39, just before the start of the influenza season (Supplementary Figure 1). We assigned all individuals with a diagnosis code for 1 or more high-risk conditions in the baseline period to the high-risk group. The remaining individuals were assigned to the not high-risk group (ie, low-risk).

### Baseline Characteristics

We measured demographic characteristics during the baseline period for each season and included age at the start of the influenza season, sex, race, ethnicity, and geographic region.

### Influenza-related Medical Encounters

The primary outcomes of interest were the incidence of influenza-related outpatient visits, ER visits, or inpatient hospitalizations. Influenza-related medical encounters included any medical encounter in which an individual had a diagnosis of influenza (ICD-9-CM codes 487.x and 488.x, along with ICD-10-CM codes J09*, J10*, and J11*). Influenza-related medical encounters were only evaluated during the influenza season. Influenza-related outpatient visits were identified using EHR and claims data. Because the EHR data used were limited to the outpatient setting, influenza-related ER visits and inpatient hospitalizations were only identified from the claims data. ER visits that occurred on the same day or on the day preceding a hospitalization were considered part of the hospitalization.

### Healthcare Resource Use and Complications

To assess prescribing patterns, we evaluated antiviral and antibiotic prescriptions following an influenza-related medical encounter (Supplementary Table 1). For outpatient and ER visits, we captured prescriptions that occurred on the day of or within 2 days after the influenza-related visit. For hospitalizations, we captured prescriptions occurring within 2 days of discharge.

To assess complications, we assessed the proportion of individuals with a subsequent medical encounter for a sinus infection or pneumonia (outpatient visit, ER visit, or hospitalization) occurring within 2 weeks of their preceding influenza-related outpatient or ER visit. Among individuals with an influenza-related hospitalization, we assessed the proportion of individuals with a pneumonia diagnosis during their influenza-related hospitalization.

Finally, to assess the severity of influenza-related hospitalizations, we measured length of stay, intensive care unit (ICU) admission, and use of mechanical ventilation. We defined length of stay as the difference between the date of admission and the date of discharge, inclusive of both.

We measured prescribing, complications, and healthcare utilization metrics for the first influenza-related outpatient visit of the season, the first influenza-related ER visit of the season, and any influenza-related hospitalization of the season.

### Data Analysis

In this descriptive analysis, we report counts and percentages for categorical variables and means and standard deviations for continuous variables. For select continuous variables, we also reported the median and interquartile range. Descriptive statistics were generated using SAS V9.4 (SAS Institute, Cary, NC). Data visualization was done in Microsoft Excel V2306 (Microsoft Corporation, Redmond, WA) and R V4.2.2 (R Foundation for Statistical Computing, Vienna, Austria).

## RESULTS

After applying the selection criteria, 887 260 individuals were included in the 2015–2016 season, 2 789 372 in 2016–2017, 3 202 455 in 2017–2018 season, 3 628 168 in 2018–2019 season, and 3 310 936 in 2019–2020 (Supplementary Table 2). Across the seasons, 37.8%–44.3% of study subjects were aged 18–49 years at the start of the season, 33.5%–38.6% were aged 50–64 years, and 17.2%–28.7% were aged ≥65 years. The percentage who were designated as high risk varied by age but was relatively consistent across seasons and included 31.8%–38.3% of individuals aged 18–49 years, 54.8%–63.0% of individuals aged 50–64 years, and 75.0%–81.4% of individuals aged ≥65 years (Supplementary Table 3).

Baseline demographic and clinical characteristics are reported by season and stratified by age group in Supplementary Table 3. The majority of individuals in all seasons and age groups were female (56.3%–64.0%), White (54.7%–71.1%), and non-Hispanic (82.7%–87.2%).

### Incidence of Medically Attended Influenza

**Figure 1.**
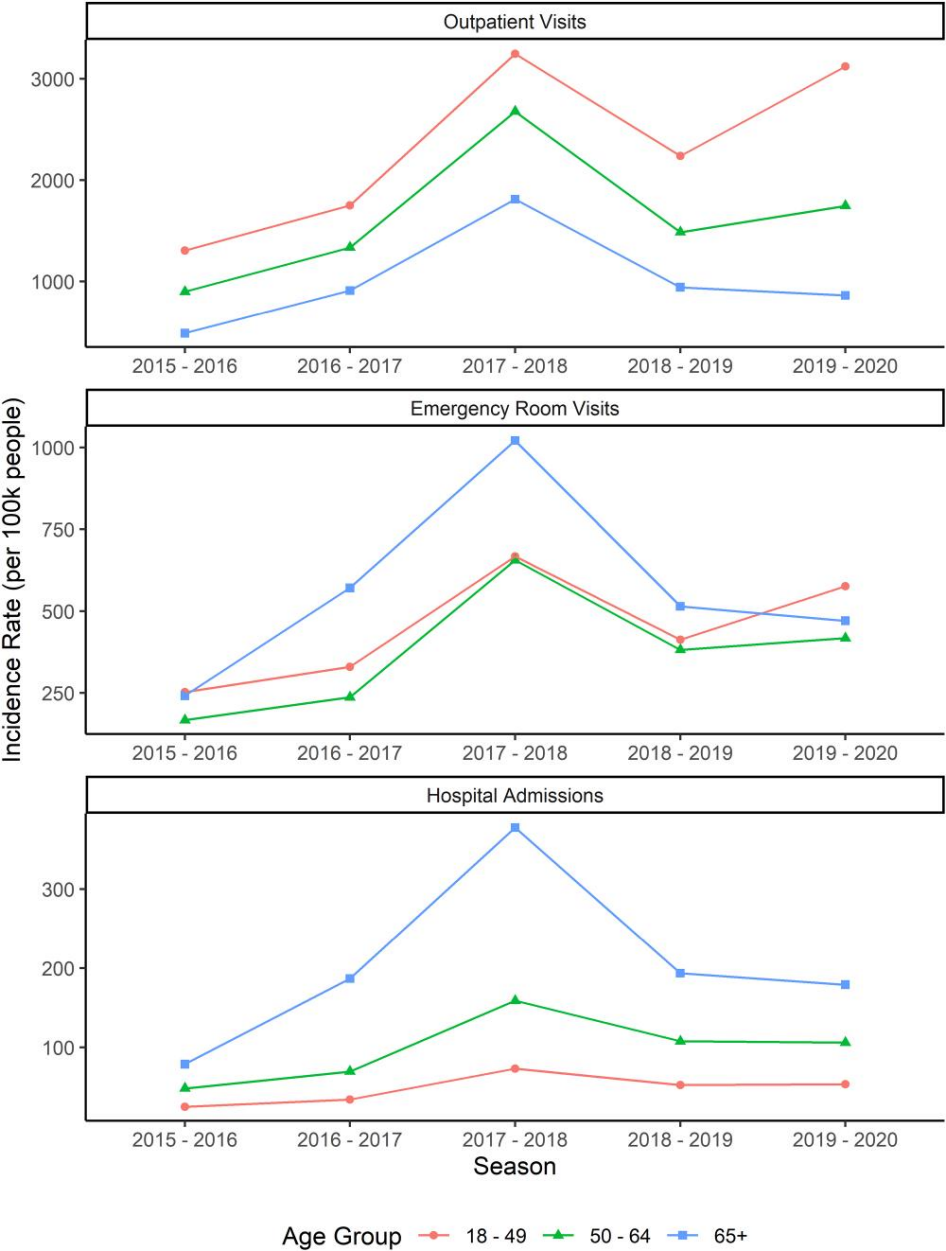
Incidence rate of outpatient visits, emergency room visits, and hospitalizations per 100 000 people by season and age group.

In the overall population, the incidence of medically attended influenza was highest in the 2017–2018 season (Figure 1). When evaluated using broader age groups, the incidence of influenza-related outpatient visits was highest among individuals aged 18–49 years (Figure 1). The median seasonal incidence of influenza-related outpatient visits per 100 000 people was 2241 in the 18–49 year age group, 1487 in the 50–64 year age group, and 910 in the ≥65 year age group. The opposite trend was observed for influenza-related hospitalizations, which were most common among individuals aged ≥65 years. The median seasonal incidence of influenza hospitalizations was 53 per 100 000 in the 18–49 year age group, 106 in the 50–64 year age group, and 187 in the ≥65 year age group. Trends in the incidence of influenza-related ER visits were less consistent across seasons but tended to be lowest among those aged 50–64 years. The median seasonal incidence of influenza-related ER visits was 413 in the 18–49 year age group, 382 in the 50–64 year age group, and 515 in the ≥65 year age group.

**Figure 2.**
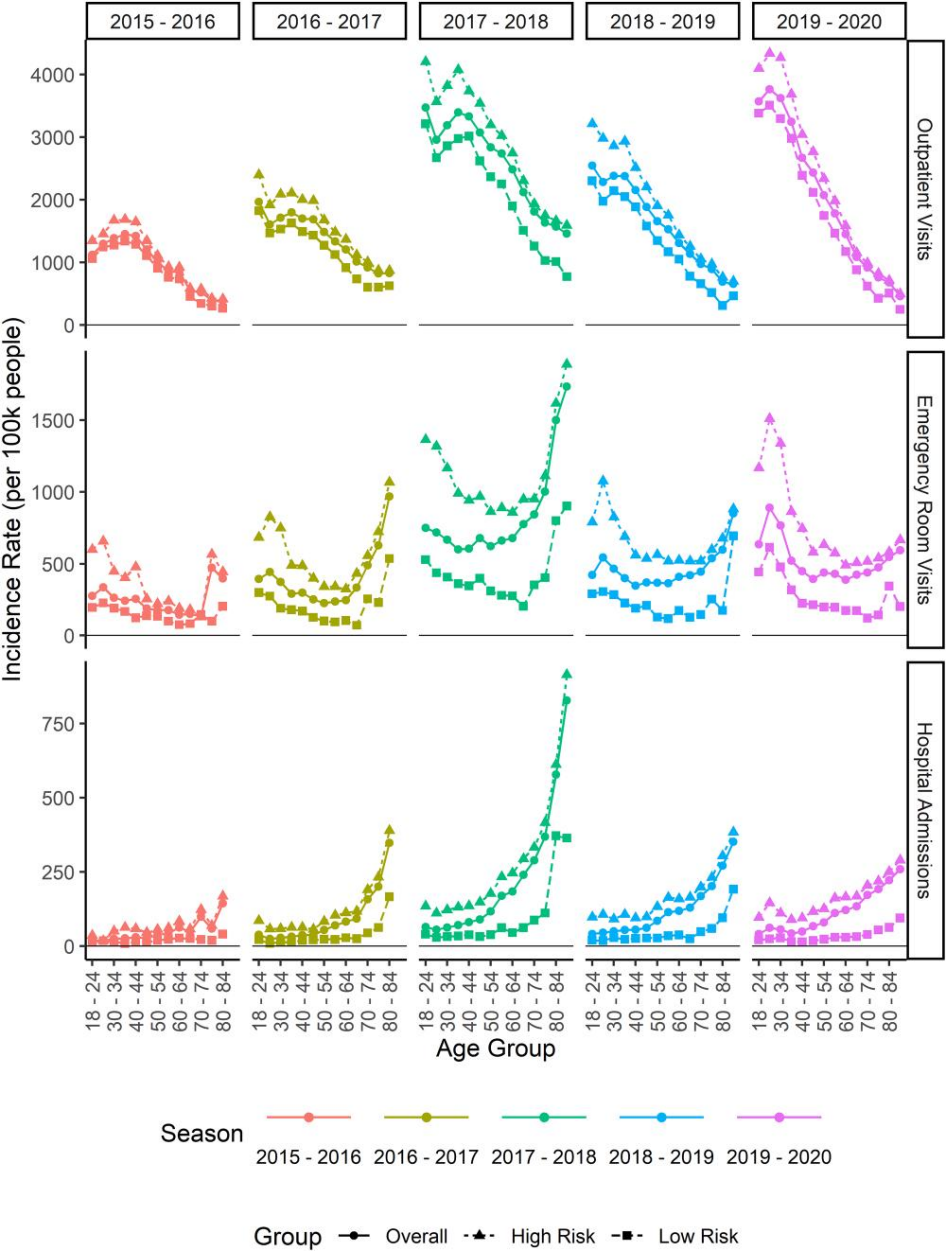
Incidence rate of outpatient visits, emergency room visits, and hospitalizations per 100 000 people in 5-year increments among adults aged ≥18 y, by season and risk status.

When evaluated in 5-year increments, the incidence of influenza-related outpatient visits was highest among people aged 18–34 years and then declined with increasing age (Figure 2). For ER visits, incidence tended to follow a U-shape, being elevated for individuals aged 18–34 years, then staying somewhat flat between ages 35 and 59 years, before increasing quickly after age 60 years. The U-shape curve for ER visits may explain the inconsistency in incidence within the broader age groups, which did not align well with the changes in incidence with age. The incidence of hospitalizations was relatively flat between aged 18–50 years and then increased with age.

Across all seasons and age groups, the incidence of medically attended influenza was higher among individuals identified as high risk than among those identified as not high risk (Figure 2). The trends across 5-year age groups described previously were observed in both the high-risk and non–high-risk populations.

### Healthcare Utilization and Complications Following Medically Attended Influenza

**Figure 3.**
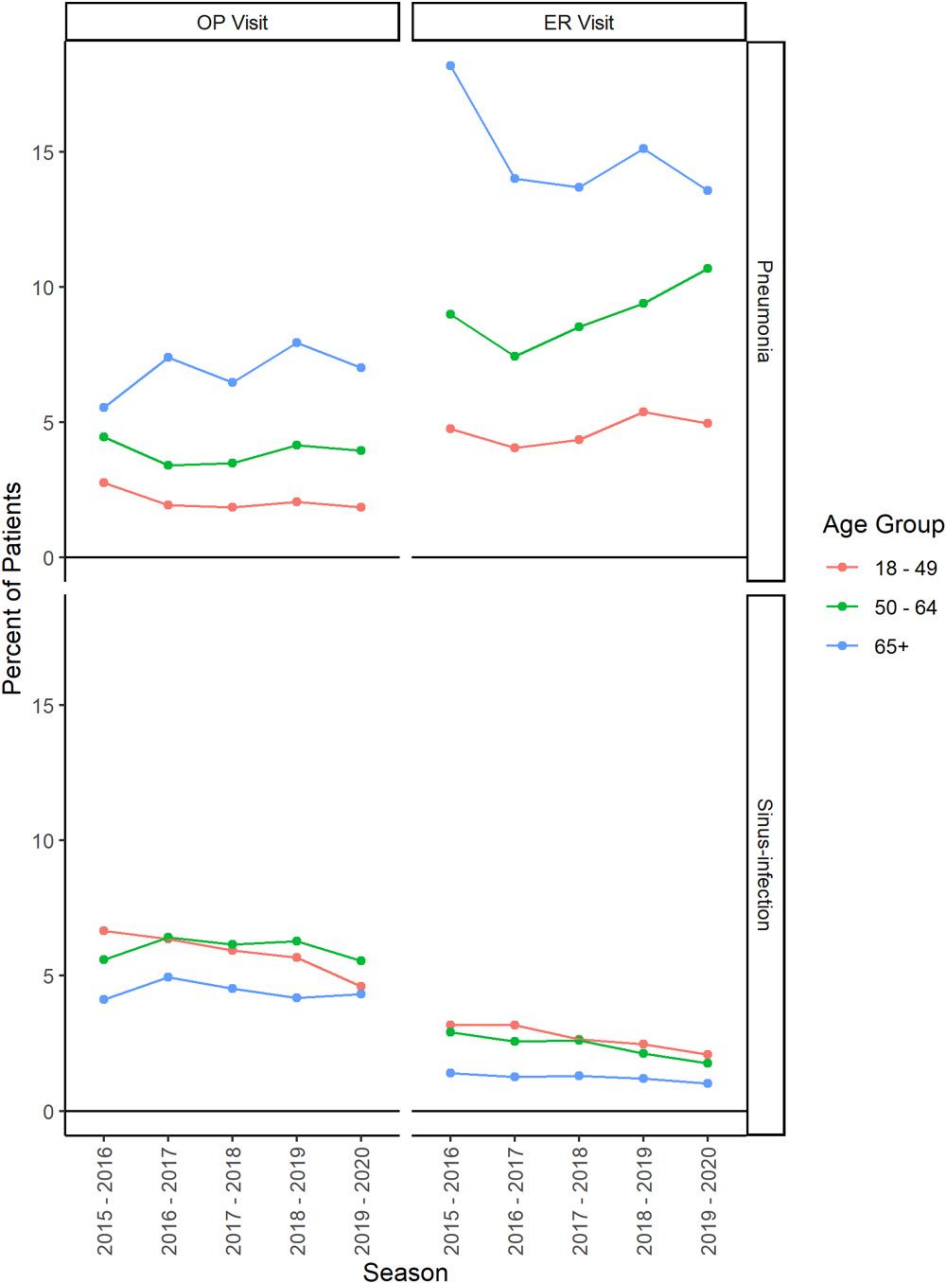
Percentage of individuals with a pneumonia-related or sinus infection–related medical encounter within 2 weeks of an influenza-related OP visit or ER visit, by season and age group. Abbreviations: ER, emergency room; OP, outpatient.

The percentage of individuals with a pneumonia-related medical encounter within 2 weeks of an influenza-related outpatient or ER visit increased with age (Figure 3). Among those with an influenza-related outpatient visit, 1.6%–2.8% of those aged 18–49 years, 3.4%–4.5% of those aged 50–64 years, and 5.5%–7.9% of those aged ≥65 years had a pneumonia-related medical encounter within 2 weeks depending on the season. Among those with an influenza-related ER visit, 4.0%–5.4% of those aged 18–49 years, 7.4%–10.7% of those aged 50–64 years, and 13.6%–18.2% of those aged ≥65 years had a pneumonia-related medical encounter within 2 weeks, depending on the season. Sinus infection–related medical encounters following an influenza-related outpatient or ER visit were more common among individuals aged 50–64 years (outpatient: 4.8%–6.4%; ER: 1.8%–2.9%) than among those aged ≥65 years (outpatient: 4.2%–4.9%; ER: 1.0%–1.4%) (Figure 3).

**Figure 4.**
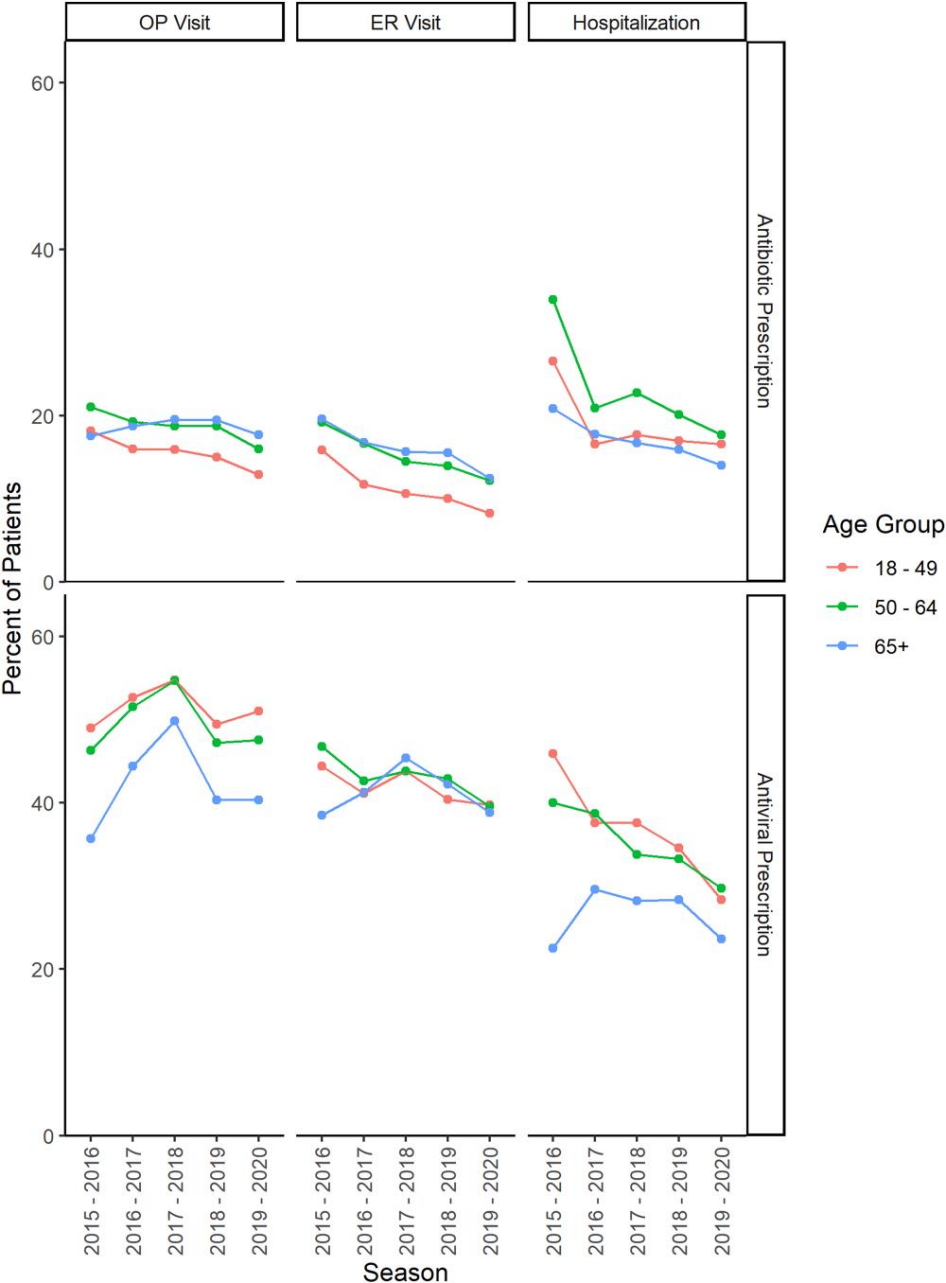
Percentage of individuals with an antiviral or antibiotic prescription filled within 2 days of an influenza-related medical encounter, by season and age group. Abbreviations: ER, emergency room; OP, outpatient.

In all age groups and seasons, antivirals were more commonly prescribed than antibiotics following an influenza-related outpatient visit, ER visit, or hospitalization (Figure 4). Antivirals tended to be more commonly prescribed to individuals aged 18–64 years compared with those aged ≥65 years following an outpatient visit or hospitalization. Antivirals also tended to be most commonly prescribed following an outpatient visit and least commonly prescribed following a hospitalization. Antibiotics were more commonly prescribed to individuals aged ≥50 years compared with those aged 18–49 years following an outpatient or ER visit. Antibiotics were more commonly prescribed following a hospitalization than following an outpatient or ER visit. There was a slight trend toward decreasing antibiotic use over the seasons, whereas antiviral use fluctuated by season.

The median length of stay for an inpatient hospitalization ranged from 3 to 5 days, with individuals aged 18–49 year having slightly shorter stays (median: 3–4 days) compared with individuals ≥65 years old (median: 5 days) (Supplementary Figure 2). Depending on the season and age group, 12.9%–20.4% of influenza-related hospitalizations included an ICU admission (Supplementary Figure 3) and 2.0%–6.6% included mechanical ventilation (Supplementary Figure 4). The median percentage of individuals with a pneumonia diagnosis across the 5 seasons was similar for all age groups: 33.2% for those aged 18–49 years, 33.6% for those aged 50–64 years (35.7%), and 34.6% for those aged ≥65 years. Hospital utilization metrics in the overall group were fairly similar across age groups.

## DISCUSSION

This study focuses on how the burden of influenza varies with age among the US adult population, as well as across individuals with and without conditions that put them at high risk for severe outcomes following an influenza infection. In general, the incidence of influenza-related outpatient visits was highest among younger adults and decreased with age, whereas the incidence of influenza-related hospitalizations increased with age beginning around age 50 years. Regardless of season or age group, around 1 in 3 individuals with an influenza-related hospitalization had pneumonia, and more than 1 in 8 had an ICU admission.

Overall, the seasonal and age-related trends observed in this analysis were consistent with the US CDC estimates, which show that influenza incidence was highest in the 2017–2018 season, and influenza-related hospitalizations among adults increased with age [1]. Although an age cut-point of 65 years old is commonly used to identify people at higher risk of severe flu outcomes, CDC data from the FluSurv-NET for the United States, a recent analysis of 7 influenza seasons in Canada, and the findings of this study suggest that hospitalization rates start to increase around age 50 years [11, 17]. Across age groups and seasons, high-risk individuals had a higher incidence of influenza-related medical encounters than not high-risk individuals. In this study, there was not a clear association between risk status and the frequency of secondary complications, as has been observed previously [18].

This retrospective study used routinely collected claims and EHR data, which can be subject to limitations, such as data entry errors and missing data. Influenza outcomes were identified based on diagnostic codes, which may not include laboratory confirmation. This study captures the incidence of medically attended influenza, but it cannot capture the incidence of influenza among those who did not seek care. There is an unknown number of individuals who have symptomatic influenza infections but do not seek care, but experience impacts such as workplace absenteeism [19]. The focus on medically attended influenza may explain the potentially counterintuitive findings that outpatient visit incidence decreased with age, whereas hospitalizations increased with age. Immunosenescence experienced by older adults can lead to a decreased likelihood of experiencing clinical signs and symptoms of an influenza infection (thus reducing their propensity to seek care following an infection) while also increasing the likelihood that an individual has more severe complications [20].

The dataset used in this analysis only included insured individuals and may not be representative of the uninsured population. Finally, the lack of any inpatient EHR data may have affected our ability to accurately identify events happening within the hospital. Data may be incomplete on drugs administered while in the hospital, such as antibiotics/antivirals given during a stay. The study's strengths include the use of a large, geographically distributed population with data sourced from both EHR and claims. This increases data completeness, as does restricting to individuals with known age, gender, and geography. Age, gender, and geography are required fields in the EHR; their presence is an indication of data quality. In addition, we repeated the analysis in 5 seasons, providing additional information on seasonal variations in the epidemiology of influenza.

Influenza epidemics in the United States are estimated to result in an average economic impact of $14.3 billion, with $4.1 billion in direct healthcare costs and $10.2 billion in indirect costs resulting from loss in productivity (numbers consumer price index adjusted to 2023 costs) [21]. Influenza vaccination is the best way to prevent influenza infections and associated healthcare resource use and complications. Despite influenza vaccination being recommended for everyone 6 months and older in the United States, there are substantial differences in coverage between age groups. Older adults aged ≥65 years receive the most focus among the adult population in terms of the burden of influenza and vaccination efforts and have the highest coverage, with 69.7% of individuals aged ≥65 years vaccinated for influenza during the 2022–2023 influenza season compared with 50.1% for individuals 50%–64% and 35.2% for those aged 18–49 years [22]. Perceived susceptibility to influenza is one of the leading drivers of influenza vaccine uptake, and younger adults are more likely to not perceive influenza as a risk [23]. More granular influenza incidence data may aid providers in communicating the burden of influenza and the benefits of influenza vaccination.

This study also demonstrated frequent antibiotic prescriptions associated with influenza-related medical encounters. Although some of the antibiotics may have been prescribed because of secondary bacterial infections, they may also include inappropriate antibiotic prescriptions. In the United States, about 30% of all antibiotics prescribed in acute care hospitals are suboptimal or unnecessary [24, 25], and vaccination is a recommended strategy for reducing antimicrobial resistance [26].

## CONCLUSIONS

This analysis demonstrates that age-associated changes in influenza incidence and care setting occur gradually with increasing age, and the incidence of hospitalizations begins to increase around 50 years of age rather than the more common cut-point of 65. Influenza infections can lead to secondary complications such as pneumonia and prescriptions for antivirals and antibiotics, underscoring the role influenza vaccination can play in combating antimicrobial resistance. Strategies to reduce the incidence and severity of influenza should consider the elevated risks among individuals aged 50–64 years in addition to the current focus on individuals aged ≥65 years.

## Supplementary Data

Supplementary materials are available at *Clinical Infectious Diseases* online. Consisting of data provided by the authors to benefit the reader, the posted materials are not copyedited and are the sole responsibility of the authors, so questions or comments should be addressed to the corresponding author.

## Supplementary Material

ciae180_Supplementary_Data

## Notes

***Author Contributions.*** I. M. and M. H. contributed to study conceptualization. All authors contributed to study design, analysis, and interpretation. K. C. and A. B. contributed to data collection and data processing. I. M. led development of the first draft of the manuscript. All authors revised the paper critically, made substantive intellectual contributions to the development of this manuscript, and approved the final version.

***Acknowledgments.*** The authors thank Mahrukh Imran (CSL Seqirus) for her contribution to the manuscript. Jessamine Winer-Jones, an employee of Veradigm, provided medical writing support for this manuscript. This support was funded by CSL Seqirus.

***Data availability.*** The data that support the findings of this study were used under license from Veradigm and Komodo Health. Due to data use agreements and their proprietary nature, restrictions apply regarding the availability of the data. Further information is available from the corresponding author.

***Financial support*.** This work was supported by CSL Seqirus.

## References

[1] Centers for Disease Control and Prevention . Disease burden of flu. 2022. Available at: https://www.cdc.gov/flu/about/burden/past-seasons.html. Accessed 13 July 2023.

[2] Martínez A , Soldevila N, Romero-Tamarit A, et al Risk factors associated with severe outcomes in adult hospitalized patients according to influenza type and subtype. PLoS One 2019; 14:e0210353. 30633778 10.1371/journal.pone.0210353 PMC6329503

[3] Cromer D , van Hoek AJ, Jit M, Edmunds WJ, Fleming D, Miller E. The burden of influenza in England by age and clinical risk group: a statistical analysis to inform vaccine policy. J Infect 2014; 68:363–71. 24291062 10.1016/j.jinf.2013.11.013

[4] Centers for Disease Control and Prevention . People at high risk of flu. 2023. Available at: https://www.cdc.gov/flu/highrisk/index.htm. Accessed 14 July 2023.

[5] Atella V , Piano Mortari A, Kopinska J, et al Trends in age-related disease burden and healthcare utilization. Aging Cell 2019; 18:e12861. 30488641 10.1111/acel.12861 PMC6351821

[6] Aw D , Silva AB, Palmer DB. Immunosenescence: emerging challenges for an ageing population. Immunology 2007; 120:435–46. 17313487 10.1111/j.1365-2567.2007.02555.x PMC2265901

[7] Castle SC , Uyemura K, Fulop T, Makinodan T. Host resistance and immune responses in advanced age. Clin Geriatr Med 2007; 23:463–79. 17631228 10.1016/j.cger.2007.03.005 PMC7135540

[8] Crooke SN , Ovsyannikova IG, Poland GA, Kennedy RB. Immunosenescence and human vaccine immune responses. Immun Ageing 2019; 16:25. 31528180 10.1186/s12979-019-0164-9 PMC6743147

[9] Crooke SN , Ovsyannikova IG, Kennedy RB, Poland GA. Immunosenescence: a systems-level overview of immune cell biology and strategies for improving vaccine responses. Exp Gerontol 2019; 124:110632. 31201918 10.1016/j.exger.2019.110632 PMC6849399

[10] Kim DK , McGeer A, Uleryk E, Coleman BL. Burden of severe illness associated with laboratory confirmed influenza in adults aged 50–64 years: a rapid review. Influenza Other Respir Viruses 2022; 16:632–42. 35044096 10.1111/irv.12955 PMC9178069

[11] Kim P , Coleman B, Kwong JC, et al Burden of severe illness associated with laboratory-confirmed influenza in adults aged 50–64 years, 2010–2011 to 2016–2017. Open Forum Infect Dis 2023; 10:ofac664. 36632417 10.1093/ofid/ofac664 PMC9830541

[12] Rolfes MA , Foppa IM, Garg S, et al Annual estimates of the burden of seasonal influenza in the United States: a tool for strengthening influenza surveillance and preparedness. Influenza Other Respir Viruses 2018; 12:132–7. 29446233 10.1111/irv.12486 PMC5818346

[13] World Medical Association . World Medical Association Declaration of Helsinki: ethical principles for medical research involving human subjects. JAMA 2013; 310:2191–4. 24141714 10.1001/jama.2013.281053

[14] Public Policy Committee, International Society of Pharmacoepidemiology . Guidelines for good pharmacoepidemiology practice (GPP). Pharmacoepidemiol Drug Saf 2016; 25:2–10. 26537534 10.1002/pds.3891

[15] Benchimol EI , Smeeth L, Guttmann A, et al The REporting of studies Conducted using Observational Routinely-collected health Data (RECORD) statement. PLoS Med 2015; 12:e1001885. 26440803 10.1371/journal.pmed.1001885 PMC4595218

[16] Boikos C , Imran M, De Lusignan S, Ortiz JR, Patriarca PA, Mansi JA. Integrating electronic medical records and claims data for influenza vaccine research. Vaccines (Basel) 2022; 10:727. 35632483 10.3390/vaccines10050727 PMC9143116

[17] Centers for Disease Control and Prevention . Influenza Hospitalization Surveillance Network (FluSurv-NET). Laboratory-confirmed influenza hospitalizations. Available at: https://gis.cdc.gov/GRASP/Fluview/FluHospRates.html. Accessed 3 August 2023.

[18] Garg S , Jain S, Dawood FS, et al Pneumonia among adults hospitalized with laboratory-confirmed seasonal influenza virus infection—United States, 2005–2008. BMC Infect Dis 2015; 15:369. 26307108 10.1186/s12879-015-1004-y PMC4550040

[19] Groenewold MR . Health-related workplace absenteeism among full-time workers—United States, 2017–18 influenza season. MMWR Morb Mortal Wkly Rep 2019; 68:577–82. 31269013 10.15585/mmwr.mm6826a1 PMC6613571

[20] Keilich SR , Bartley JM, Haynes L. Diminished immune responses with aging predispose older adults to common and uncommon influenza complications. Cell Immunol 2019; 345:103992. 31627841 10.1016/j.cellimm.2019.103992 PMC6939636

[21] Putri WCWS , Muscatello DJ, Stockwell MS, Newall AT. Economic burden of seasonal influenza in the United States. Vaccine 2018; 36:3960–6. 29801998 10.1016/j.vaccine.2018.05.057

[22] Centers for Disease Control and Prevention . Flu vaccination coverage, United States, 2022–23 influenza season. 2023. Available at: https://www.cdc.gov/flu/fluvaxview/coverage-2223estimates.htm. Accessed 26 October 2023.

[23] Nowak GJ , Sheedy K, Bursey K, Smith TM, Basket M. Promoting influenza vaccination: insights from a qualitative meta-analysis of 14 years of influenza-related communications research by U.S. Centers for Disease Control and Prevention (CDC). Vaccine 2015; 33:2741–56. 25936726 10.1016/j.vaccine.2015.04.064 PMC5856146

[24] Dellit TH , Owens RC, McGowan JE, et al Infectious Diseases Society of America and the Society for Healthcare Epidemiology of America guidelines for developing an institutional program to enhance antimicrobial stewardship. Clin Infect Dis 2007; 44:159–77. 17173212 10.1086/510393

[25] Fridkin S , Baggs J, Fagan R, et al Vital signs: improving antibiotic use among hospitalized patients. Morb Mortal Wkly Rep 2014; 63:194–200. PMC4584728 24598596

[26] van Heuvel , Paget J, Dückers M, Caini S. The impact of influenza and pneumococcal vaccination on antibiotic use: an updated systematic review and meta-analysis. Antimicrobial resistance and infection control 2023; 12:70–NaN. 37452389 10.1186/s13756-023-01272-6 PMC10347879

