# Supplementary material for: Incidence of Influenza-related Medical Encounters and the Associated Healthcare Resource Use and Complications Across Adult Age Groups in the United States During the 2015–2020 Influenza Seasons
Source: Clin Infect Dis. 2024 Apr 3;79(3):778–86. doi: 10.1093/cid/ciae180 (PMC11426264; doi:10.1093/cid/ciae180)
Supplement: ciae180_Supplementary_Data [file ciae180_supplementary_data.docx]

Supplementary Figure 1. Study time periods, example of 2015–2016 Season

12 Month Baseline Period

Start Week 40

2015

Start Week 40

2014

End Week 20

2016

Off-season period

Weeks 21-39

Influenza Season

Supplemental Figure 2: Mean and median hospitalization length of stay in days, by season, risk status, and age group


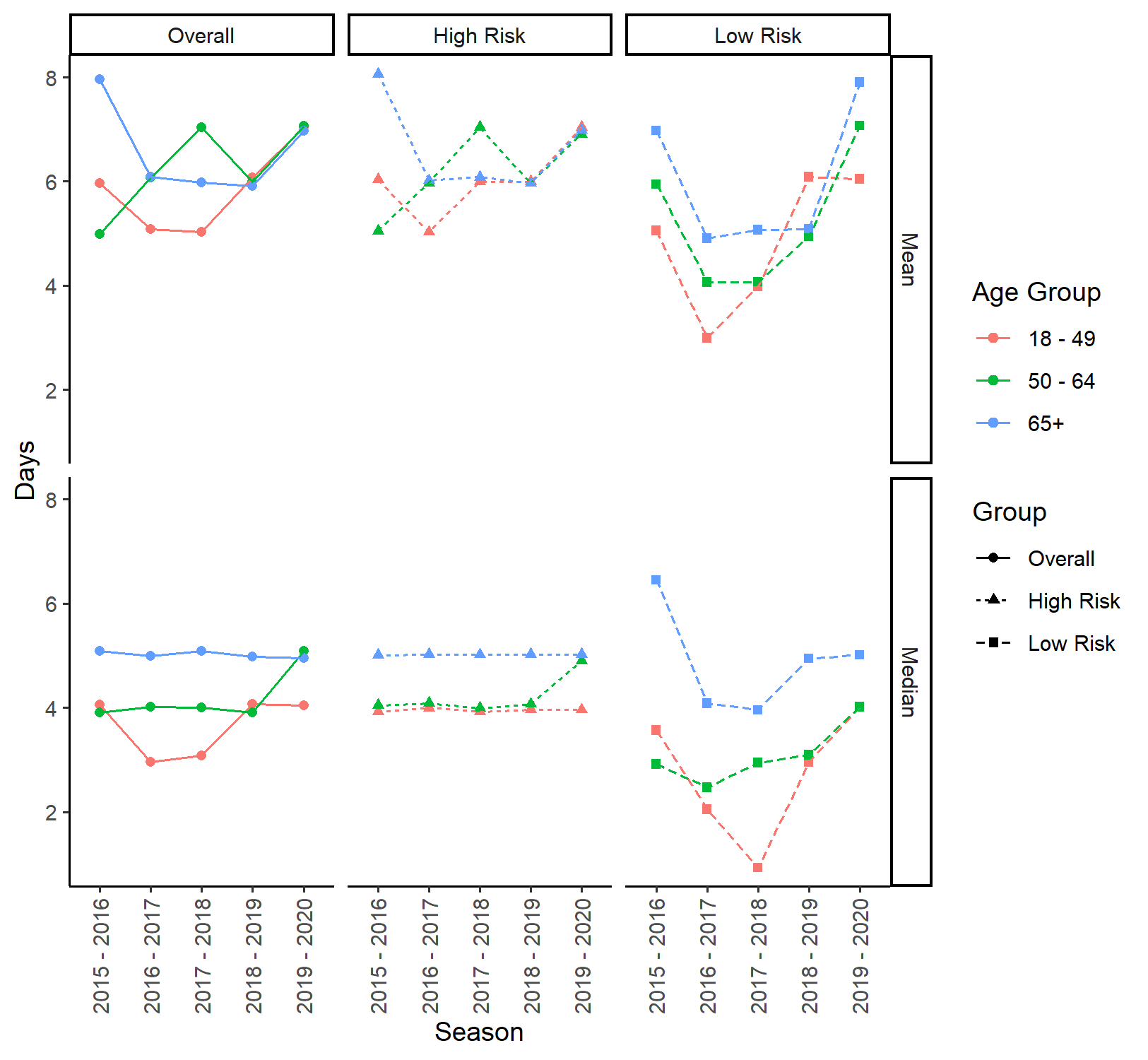


Supplemental Figure 3: Percentage of hospitalized individuals with an intensive care unit admission, by season, risk status, and age group


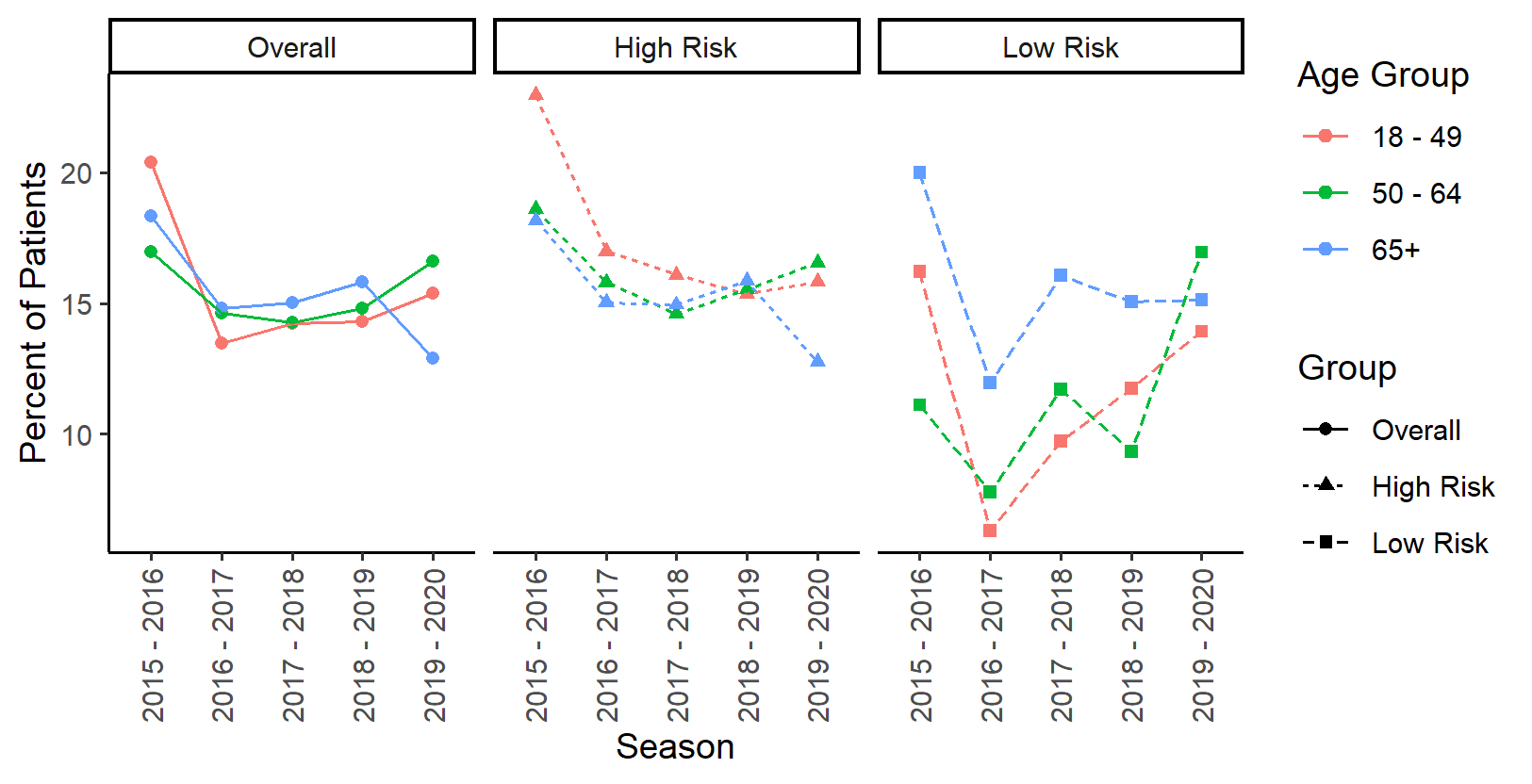


Supplemental Figure 4: Percentage of hospitalized individuals with mechanical ventilation, by season, risk status, and age group


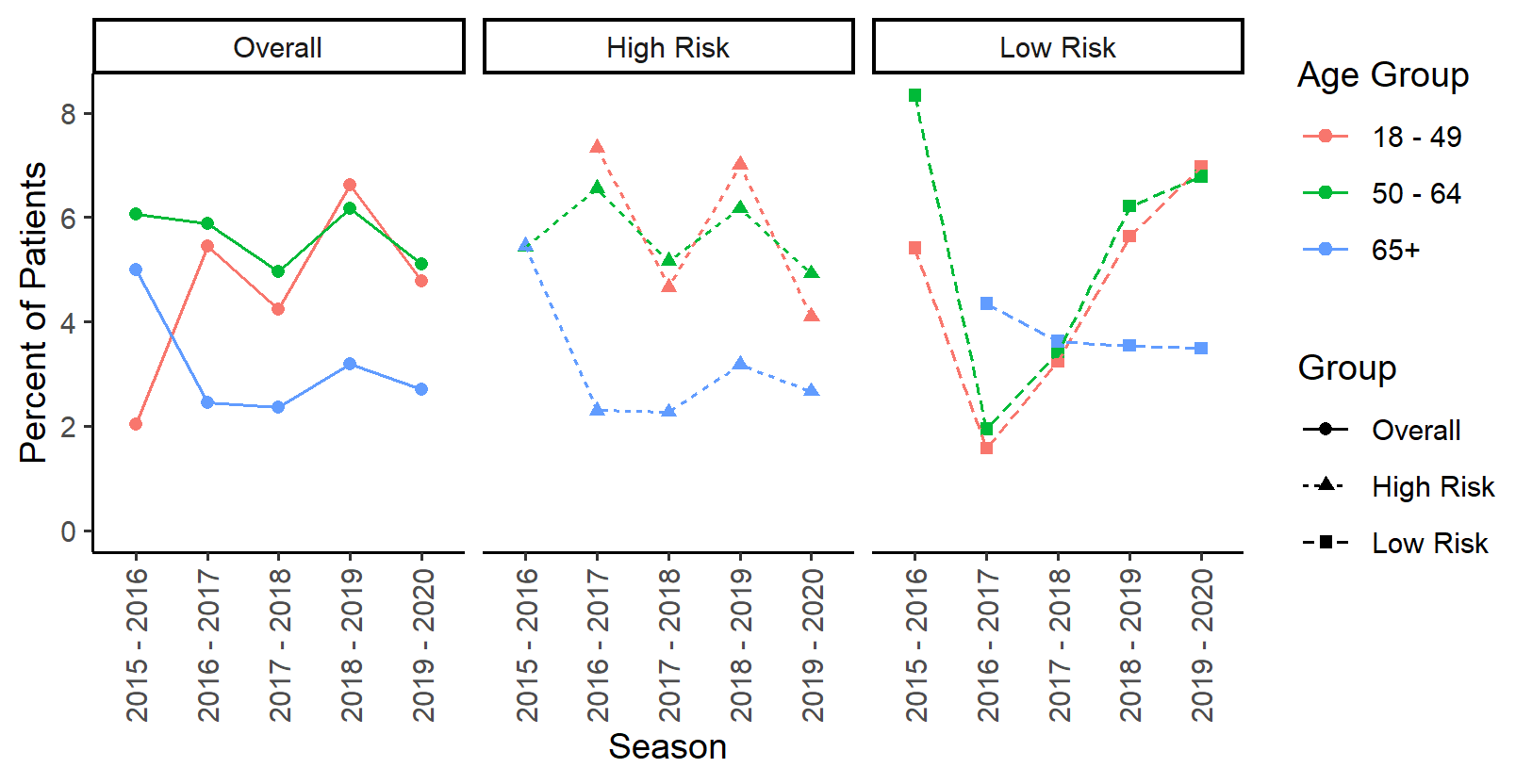


Supplementary Figure 5: Percent of hospitalized individuals with a pneumonia diagnosis during their influenza hospitalization, by season, risk status, and age group


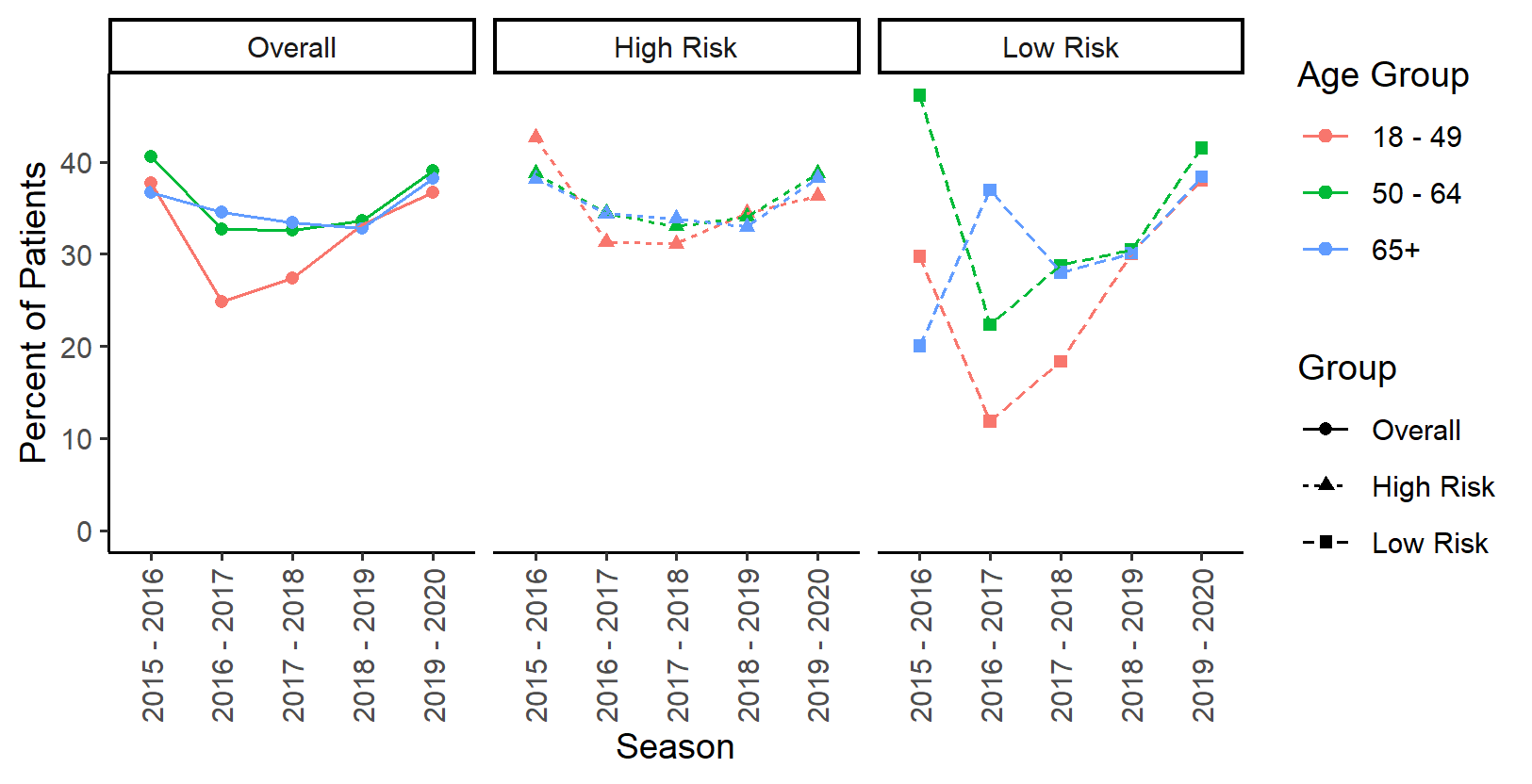


Supplementary Table 1. Study measures

| Influenza risk factors | - Asthma - Neurologic and neurodevelopmental conditions - Blood disorders (e.g., sickle cell disease) - Chronic lung disease (e.g., chronic obstructive pulmonary disease, cystic fibrosis) - Endocrine disorders (e.g., diabetes mellitus) - Heart disease (e.g., congenital heart disease, congestive heart failure, coronary artery disease) - Kidney diseases - Liver disorders - Metabolic disorders (e.g., inherited metabolic disorders, mitochondrial disorders) - Obesity (body mass index of 40 or higher) - Weakened immune system due to disease (e.g., HIV or AIDS, leukemia) or medications (e.g., chemotherapy or radiation treatment for cancer, chronic corticosteroids) - Stroke |
| --- | --- |
| Antivirals | - baloxavir - peramivir - oseltamivir - zanamivir |
| Antibiotics | - amoxicillin - azithromycin - clarithromycin - clavulanate - doxycycline - erythromycin - levofloxacin |
| Demographic characteristics | - Age at Start of Season - Sex (Female, Male) - Race (Black or African American, White, Other, Not Reported) - Ethnicity (Hispanic, Non-Hispanic, Not Reported) - Geographic region (Northeast, Midwest, South, West, Other) |

Supplementary Table 2: Study subject selection by season

|  | **2015–2016** | **2016–2017** | **2017–2018** | **2018–2019** | **2019–2020** |
| --- | --- | --- | --- | --- | --- |
| 1) Individual had a transcript record in the Veradigm EMR during the influenza season | 34,545,275 | 33,699,265 | 32,825,827 | 29,136,217 | 23,999,757 |
| 2) Individual had a transcript record in the Veradigm EMR at least 1 year prior to start of influenza season | 19,601,796 (56.7%) | 21,736,823 (64.5%) | 22,152,225 (67.5%) | 20,637,829 (70.8%) | 18,033,597 (75.1%) |
| 3) Individual was continuously enrolled in the claims data from at least 1 year prior to the start of the influenza season to 120 days after the end of the influenza season | 1,354,241 (6.9%) | 4,105,800 (18.9%) | 4,517,236 (20.4%) | 4,805,530 (23.3%) | 4,268,586 (23.7%) |
| 4) Individual did not have an influenza-related medical encounter in weeks 21-39 prior to the start of the influenza season | 1,351,834 (99.8%) | 4,099,890 (99.9%) | 4,509,829 (99.8%) | 4,796,370 (99.8%) | 4,257,243 (99.7%) |
| 5) Individual was at least 18 years old at start of influenza season | 1,118,466 (82.7%) | 3,410,121 (83.2%) | 3,768,633 (83.6%) | 4,130,019 (86.1%) | 3,700,784 (86.9%) |
| 6) Individual did not have missing gender and geographic region data | 887,260 (79.3%) | 2,789,372 (81.8%) | 3,202,455 (85.0%) | 3,628,168 (87.8%) | 3,310,936 (89.5%) |
| Individuals aged 18 - 49 | 392,647 (44.3%) | 1,130,716 (40.5%) | 1,285,802 (40.2%) | 1,408,171 (38.8%) | 1,252,459 (37.8%) |
| Individuals aged 50 - 64 | 342,439 (38.6%) | 1,003,938 (36.0%) | 1,129,708 (35.3%) | 1,249,587 (34.4%) | 1,108,895 (33.5%) |
| Individuals aged 65+ | 152,174 (17.2%) | 654,718 (23.5%) | 786,945 (24.6%) | 970,410 (26.7%) | 949,582 (28.7%) |

Supplementary Table 3. Characteristics of Individuals Included in (A) the 2015–2016 Season, (B) the 2016–2017 Season, (C) the 2017–2018 Season, (D) the 2018–2019 Season, and (E) the 2019–2020 Season.

| Supplementary Table 3A: 2015–2016 Season | | | |
| --- | --- | --- | --- |
|  | 18–49 Yrs | 50–64 Yrs | ≥65 Yrs |
|  | N = 392,647 | N = 342,439 | N = 152,174 |
| Age, mean (SD) | 35.6 (9.3) | 57.0 (4.1) | 72.3 (5.8) |
| Age, median (IQR) | 37 (28 - 44) | 57 (53 - 61) | 71 (67 - 77) |
| Female sex, N (%) | 251,269 (64.0%) | 200,664 (58.6%) | 86,988 (57.2%) |
| Race, N (%) |  |  |  |
| Asian | 10,883 (2.8%) | 7,418 (2.2%) | 3,789 (2.5%) |
| Black or African American | 32,235 (8.2%) | 26,024 (7.6%) | 11,046 (7.3%) |
| Other | 14,042 (3.6%) | 10,874 (3.2%) | 5,924 (3.9%) |
| Unknown | 81,602 (20.8%) | 56,450 (16.5%) | 22,291 (14.6%) |
| White | 253,885 (64.7%) | 241,673 (70.6%) | 109,124 (71.7%) |
| Ethnicity, N (%) |  |  |  |
| Hispanic | 33,367 (8.5%) | 19,956 (5.8%) | 10,525 (6.9%) |
| Non-Hispanic | 324,890 (82.7%) | 294,766 (86.1%) | 130,965 (86.1%) |
| Unknown/not reported | 34,390 (8.8%) | 27,717 (8.1%) | 10,684 (7.0%) |
| Geographic region, N (%) |  |  |  |
| Northeast | 90,810 (23.1%) | 85,305 (24.9%) | 46,589 (30.6%) |
| Midwest | 91,374 (23.3%) | 79,049 (23.1%) | 34,638 (22.8%) |
| South | 146,816 (37.4%) | 125,720 (36.7%) | 50,400 (33.1%) |
| West | 63,644 (16.2%) | 52,351 (15.3%) | 20,492 (13.5%) |
| Other/unknown | 3 (0.0%) | 14 (0.0%) | 55 (0.0%) |
| High-risk, N (%) | 125,008 (31.8%) | 187,827 (54.8%) | 114,095 (75.0%) |

BMI, body mass index; ER, emergency room; IQR, interquartile range; OP, outpatient; SD, standard deviation;

| Supplementary Table 3B: 2016–2017 Season | | | |
| --- | --- | --- | --- |
|  | 18–49 Yrs | 50–64 Yrs | ≥65 Yrs |
|  | N = 1,130,716 | N = 1,003,938 | N = 654,718 |
| Age, mean (SD) | 35.7 (9.4) | 57.1 (4.2) | 73.6 (6.1) |
| Age, median (IQR) | 37 (28 - 44) | 57 (54 - 61) | 73 (68 - 79) |
| Female sex, N (%) | 719,706 (63.7%) | 578,766 (57.6%) | 376,989 (57.6%) |
| Race, N (%) |  |  |  |
| Asian | 35,622 (3.2%) | 25,482 (2.5%) | 15,340 (2.3%) |
| Black or African American | 96,459 (8.5%) | 81,325 (8.1%) | 48,707 (7.4%) |
| Other | 42,332 (3.7%) | 34,020 (3.4%) | 22,477 (3.4%) |
| Unknown | 257,403 (22.8%) | 187,414 (18.7%) | 104,048 (15.9%) |
| White | 698,900 (61.8%) | 675,697 (67.3%) | 464,146 (70.9%) |
| Ethnicity, N (%) |  |  |  |
| Hispanic | 110,919 (9.8%) | 70,280 (7.0%) | 45,245 (6.9%) |
| Non-Hispanic | 935,584 (82.7%) | 862,044 (85.9%) | 569,819 (87.0%) |
| Unknown/not reported | 84,213 (7.4%) | 71,614 (7.1%) | 39,654 (6.1%) |
| Geographic region, N (%) |  |  |  |
| Northeast | 278,216 (24.6%) | 271,663 (27.1%) | 231,500 (35.4%) |
| Midwest | 247,229 (21.9%) | 214,486 (21.4%) | 119,471 (18.2%) |
| South | 384,875 (34.0%) | 335,925 (33.5%) | 204,364 (31.2%) |
| West | 219,500 (19.4%) | 180,905 (18.0%) | 98,089 (15.0%) |
| Other/unknown | 896 (0.1%) | 959 (0.1%) | 1,294 (0.2%) |
| High-risk, N (%) | 408,274 (36.1%) | 586,362 (58.4%) | 507,004 (77.4%) |

BMI, body mass index; ER, emergency room; IQR, interquartile range; OP, outpatient; SD, standard deviation;

| Supplementary Table 3C: 2017–2018 Season | | | |
| --- | --- | --- | --- |
|  | 18–49 Yrs | 50–64 Yrs | ≥65 Yrs |
|  | N = 1,285,802 | N = 1,129,708 | N = 786,945 |
| Age, mean (SD) | 35.6 (9.3) | 57.2 (4.2) | 73.8 (6.3) |
| Age, median (IQR) | 37 (28 - 44) | 57 (54 - 61) | 73 (68 - 79) |
| Female sex, N (%) | 816,181 (63.5%) | 649,522 (57.5%) | 452,568 (57.5%) |
| Race, N (%) |  |  |  |
| Asian | 43,779 (3.4%) | 31,323 (2.8%) | 19,871 (2.5%) |
| Black or African American | 109,989 (8.6%) | 94,943 (8.4%) | 60,363 (7.7%) |
| Other | 50,618 (3.9%) | 40,960 (3.6%) | 28,154 (3.6%) |
| Unknown | 330,747 (25.7%) | 242,602 (21.5%) | 144,442 (18.4%) |
| White | 750,669 (58.4%) | 719,880 (63.7%) | 534,115 (67.9%) |
| Ethnicity, N (%) |  |  |  |
| Hispanic | 130,697 (10.2%) | 86,925 (7.7%) | 57,256 (7.3%) |
| Non-Hispanic | 1,070,926 (83.3%) | 968,336 (85.7%) | 685,953 (87.2%) |
| Unknown/not reported | 84,179 (6.5%) | 74,447 (6.6%) | 43,736 (5.6%) |
| Geographic region, N (%) |  |  |  |
| Northeast | 321,692 (25.0%) | 315,725 (27.9%) | 272,540 (34.6%) |
| Midwest | 268,483 (20.9%) | 223,731 (19.8%) | 137,859 (17.5%) |
| South | 430,010 (33.4%) | 373,975 (33.1%) | 251,969 (32.0%) |
| West | 264,576 (20.6%) | 215,114 (19.0%) | 122,582 (15.6%) |
| Other/unknown | 1,041 (0.1%) | 1,163 (0.1%) | 1,995 (0.3%) |
| High-risk, N (%) | 492,585 (38.3%) | 711,789 (63.0%) | 640,643 (81.4%) |

BMI, body mass index; ER, emergency room; IQR, interquartile range; OP, outpatient; SD, standard deviation;

| Supplementary Table 3D: 2018–2019 Season | | | |
| --- | --- | --- | --- |
|  | 18–49 Yrs | 50–64 Yrs | ≥65 Yrs |
|  | N = 1,408,171 | N = 1,249,587 | N = 970,410 |
| Age, mean (SD) | 35.8 (9.2) | 57.3 (4.2) | 73.8 (6.5) |
| Age, median (IQR) | 37 (28 - 44) | 57 (54 - 61) | 72 (68 - 79) |
| Female sex, N (%) | 895,206 (63.6%) | 716,458 (57.3%) | 551,393 (56.8%) |
| Race, N (%) |  |  |  |
| Asian | 45,755 (3.2%) | 34,134 (2.7%) | 24,366 (2.5%) |
| Black or African American | 118,835 (8.4%) | 107,395 (8.6%) | 74,445 (7.7%) |
| Other | 57,523 (4.1%) | 47,462 (3.8%) | 36,448 (3.8%) |
| Unknown | 392,927 (27.9%) | 291,216 (23.3%) | 189,375 (19.5%) |
| White | 793,131 (56.3%) | 769,380 (61.6%) | 645,776 (66.5%) |
| Ethnicity, N (%) |  |  |  |
| Hispanic | 142,660 (10.1%) | 99,397 (8.0%) | 72,393 (7.5%) |
| Non-Hispanic | 1,174,551 (83.4%) | 1,066,693 (85.4%) | 842,306 (86.8%) |
| Unknown/not reported | 90,960 (6.5%) | 83,497 (6.7%) | 55,711 (5.7%) |
| Geographic region, N (%) |  |  |  |
| Northeast | 314,105 (22.3%) | 310,861 (24.9%) | 280,522 (28.9%) |
| Midwest | 251,670 (17.9%) | 219,050 (17.5%) | 157,137 (16.2%) |
| South | 550,428 (39.1%) | 476,941 (38.2%) | 363,728 (37.5%) |
| West | 291,103 (20.7%) | 241,376 (19.3%) | 165,243 (17.0%) |
| Other/unknown | 865 (0.1%) | 1,359 (0.1%) | 3,780 (0.4%) |
| High-risk, N (%) | 531,287 (37.7%) | 768,957 (61.5%) | 773,005 (79.7%) |

BMI, body mass index; ER, emergency room; IQR, interquartile range; OP, outpatient; SD, standard deviation

| Supplementary Table 3E: 2019–2020 Season | | | |
| --- | --- | --- | --- |
|  | 18–49 Yrs | 50–64 Yrs | ≥65 Yrs |
|  | N = 1,252,459 | N = 1,108,895 | N = 949,582 |
| Age, mean (SD) | 35.8 (9.2) | 57.3 (4.2) | 74.0 (6.7) |
| Age, median (IQR) | 37 (29 - 44) | 58 (54 - 61) | 73 (68 - 79) |
| Female sex, N (%) | 793,082 (63.3%) | 631,525 (57.0%) | 534,725 (56.3%) |
| Race, N (%) |  |  |  |
| Asian | 40,473 (3.2%) | 30,132 (2.7%) | 22,870 (2.4%) |
| Black or African American | 99,899 (8.0%) | 91,212 (8.2%) | 68,369 (7.2%) |
| Other | 51,973 (4.1%) | 43,119 (3.9%) | 35,896 (3.8%) |
| Unknown | 375,256 (30.0%) | 277,078 (25.0%) | 196,517 (20.7%) |
| White | 684,858 (54.7%) | 667,354 (60.2%) | 625,930 (65.9%) |
| Ethnicity, N (%) |  |  |  |
| Hispanic | 120,864 (9.7%) | 88,842 (8.0%) | 70,471 (7.4%) |
| Non-Hispanic | 1,050,450 (83.9%) | 944,367 (85.2%) | 823,911 (86.8%) |
| Unknown/not reported | 81,145 (6.5%) | 75,686 (6.8%) | 55,200 (5.8%) |
| Geographic region, N (%) |  |  |  |
| Northeast | 282,528 (22.6%) | 280,434 (25.3%) | 264,321 (27.8%) |
| Midwest | 193,875 (15.5%) | 165,714 (14.9%) | 140,746 (14.8%) |
| South | 514,409 (41.1%) | 445,452 (40.2%) | 376,151 (39.6%) |
| West | 261,388 (20.9%) | 216,331 (19.5%) | 163,484 (17.2%) |
| Other/unknown | 259 (0.0%) | 964 (0.1%) | 4,880 (0.5%) |
| High-risk, N (%) | 475,259 (37.9%) | 684,762 (61.8%) | 761,366 (80.2%) |

BMI, body mass index; ER, emergency room; IQR, interquartile range; OP, outpatient; SD, standard deviation
